# Supplementary material for: Characterization of the transcriptome and EST-SSR development in Boea clarkeana, a desiccation-tolerant plant endemic to China
Source: PeerJ. 2017 Jun 15;5:e3422. doi: 10.7717/peerj.3422 (PMC5474092; doi:10.7717/peerj.3422)
Supplement: Table S1 [file peerj-05-3422-s001.docx]

**Supplemental Information**

**Supplemental Tables**

**Table S1 GO classification of 17 SSR-containing ESTs.**

| **Unigene^*^** | **GO** | | |
| --- | --- | --- | --- |
|  | **biological_process** | **cellular_component** | **molecular_function** |
| BC1 | - | cell | - |
|  |  | cell part |  |
|  |  | extracellular region |  |
|  |  | membrane |  |
|  |  | organelle |  |
|  |  | organelle part |  |
| BC2 | response to stimulus | - | - |
| BC3 | - | - | - |
| BC4 | biological regulation | - | binding |
|  | cellular process |  | catalytic activity |
|  | metabolic process |  | molecular transducer activity |
|  | regulation of biological process |  |  |
|  | response to stimulus |  |  |
|  | signaling |  |  |
|  | single-organism process |  |  |
| BC5 | - | - | - |
| BC6 | - | - | - |
| BC7 | metabolic process | cell | - |
|  | single-organism process | cell part |  |
| BC8 | biological regulation | cell | binding |
|  | cellular process | cell junction | catalytic activity |
|  | establishment of localization | membrane | molecular transducer activity |
|  | cellular component organization or biogenesis | organelle | structural molecule activity |
|  | localization |  |  |
|  | metabolic process |  |  |
|  | multicellular organismal process |  |  |
|  | negative regulation of biological process |  |  |
|  | regulation of biological process |  |  |
|  | response to stimulus |  |  |
|  | signaling |  |  |
|  | single-organism process |  |  |
| BC9 | - | - | binding |
|  |  |  | catalytic activity |
| BC10 | cellular process, metabolic process | cell | catalytic activity |
|  |  | cell junction |  |
|  |  | organelle |  |
|  |  | metabolic process |  |
| BC11 | - | - | - |
| BC12 | biological regulation | cell | binding |
|  | cellular process | cell part | protein binding transcription factor activity |
|  | developmental process | organelle |  |
|  | metabolic process |  |  |
|  | multicellular organismal process |  |  |
|  | regulation of biological process |  |  |
|  | reproduction |  |  |
|  | reproductive process |  |  |
|  | response to stimulus |  |  |
|  | single-organism process |  |  |
| BC13 | - | - | - |
| BC14 | - | - | - |
| BC15 | biological regulation | cell | binding |
|  | cellular component organization or biogenesis | cell part | catalytic activity |
|  | cellular process | membrane |  |
|  | growth,metabolic process | organelle |  |
|  | multi-organism process | organelle part |  |
|  | negative regulation of biological process |  |  |
|  | regulation of biological process |  |  |
|  | response to stimulus |  |  |
|  | single-organism process |  |  |
| BC16 | - | - | - |
| BC17 | - | - | - |

*Note:*^*^ The name of each unigene is replaced with the name of the EST-SSR marker it contains.
